# Anticholinergic Medication and Caries Status Predict Xerostomia under 65

**DOI:** 10.3390/dj11040087

**Published:** 2023-03-23

**Authors:** Hui Ling Cheah, Michael Gray, Shahenda Aboelmagd, Abdul Basir Barmak, Szilvia Arany

**Affiliations:** 1 General Dentistry, Department of Dentistry, Eastman Institute of Oral Health, University of Rochester, Rochester, NY 14620, USA; 2 Specialty Care Clinic, Eastman Institute of Oral Health, University of Rochester, Rochester, NY 14620, USA

**Keywords:** xerostomia, anticholinergic, medication, caries, prediction

## Abstract

The use of anticholinergic medications is increasing in younger ages, yet information about xerostomia, the most common anticholinergic side effect, is limited. This case–control retrospective study examines the relationship between anticholinergic medication-induced xerostomia and caries status among adults between 18 and 65 years of age. The study sample comprised 649 cases with xerostomia and 649 age- and gender-matched controls. The anticholinergic burden was estimated using the anticholinergic drug scale (ADS). Caries experience was recorded by calculating the Decayed, Missing, Filled Tooth (DMFT) index. Individuals with xerostomia had a higher mean DMFT index (16.02 ± 9.50), which corresponded with a higher level of anticholinergic exposure from medications (3.26 ± 2.81) compared to their age and gender-matched controls without xerostomia (13.83 + 8.83 and 1.89 ± 2.45, respectively). Logistic regression analysis verified the effects of DMFT, the total number of AC medications, and the ADS burden on xerostomia status. Comparing adults with or without xerostomia revealed statistical differences in several risk factors, such as smoking, diabetes, sleep apnea, and the utilization of anticholinergic medications. A personalized dental care plan should include the evaluation of the anticholinergic burden from medications regardless of the patient’s age to prevent increased caries severity.

## 1. Introduction

Xerostomia and salivary gland dysfunction can be associated with the consumption of medications, salivary gland diseases, autoimmune diseases (primary and secondary Sjogren’s syndrome), psychological factors, radiation, and chemotherapy [1]. The subjective sensation of dry mouth is xerostomia, a symptom that the patients perceive [2], and it may or may not be accompanied by hyposalivation [3]. According to a systematic review and meta-analysis, the overall prevalence of xerostomia among all age groups is estimated to be 23% [4]. In adults aged 20–59 years, a prospective study reported 11% of xerostomia prevalence [5].

Saliva secretion covers the intraoral tissues and eliminates food debris and bacteria by swallowing. Decreased saliva secretion and xerostomia commonly manifest in speaking, swallowing, and chewing difficulty [6,7,8]. Intraoral dryness contributes to oral discomfort, altered taste, increased risk of oral infections, and substantially reduced quality of life [9,10,11]. Previous clinical studies have shown that reduced saliva flow increases the occurrence of caries. In xerostomia patients, carious lesions frequently have a rapid onset and progression, despite good oral hygiene [12]. Consequently, the higher tendency to rampant, recurrent caries may lead to early tooth loss.

Drug-induced xerostomia has been attributed to a wide range of mechanisms. The most commonly accepted mechanism is that medications block the actions of muscarinic receptors in the salivary glands [13]. Polypharmacy, defined as the regular use of at least five medications, is widely known as the primary cause of xerostomia, affecting one in five patients in the United States [14]. Polypharmacy is most commonly reported in adults over 65 years. In addition, medication-induced xerostomia and its destructive effects on oral health are almost exclusively described in the context of older adults [15]. Increasing usage of multiple medications in younger rather than older adult age groups [16] brought greater attention to polypharmacy-related issues. An increasing number of young adults are exposed to polypharmacy in various diseases such as respiratory, mental health, cardiometabolic, endocrinological, osteometabolic, and chronic pain [17]. Younger adults diagnosed with primary headaches are also exposed to high polypharmacy comparable to the older age groups [18]. Combining multiple anticholinergics is more likely to induce xerostomia [19]. However, data for medication-induced xerostomia in adults younger than 50 years of age are rare to encounter.

More than 600 drugs have anticholinergic properties [20]. Most commonly prescribed medications with significant anticholinergic activity as unwanted side effects include antihistamines, antispasmodics, antidepressants, and antipsychotics. Prolonged exposure to commonly prescribed medications confers a detectable anticholinergic burden [21] on the body. Anticholinergic burden measured [22] using anticholinergic scales estimates the cumulative effect of taking one or more drugs capable of causing the anticholinergic adverse effect. The anticholinergic drug score (ADS) [23] expressed the overall anticholinergic impact of medications and was used in dental studies previously [24,25].

The purpose of this study is to gain information about anticholinergics drug use among non-geriatric adults. The aim of the present study was to retrospectively compare the caries status of young- and middle-aged individuals with and without anticholinergic medication-induced xerostomia.

## 2. Methods

The study protocol was approved by the University of Rochester Research Subjects Review Board (RSRB No. 00003301, 19 February 2019). The study was planned in accordance with the Declaration of Helsinki and the United States Federal Policy for the Protection of Human Subjects. Adult patients from 18 to 65 years who received a dental examination at the Department of Dentistry, Eastman Institute for Oral Health, Rochester, NY, between April 2010 and January 2019 were eligible to be chosen for the study in the case and control groups. Accordingly, individuals with self-reported xerostomia represented the cases, and all controls were matched by age and gender to cases. Four investigators reviewed the electronic medical (eRecord @Epic Systems Corporation, Verona, Italy) and dental records (axiUm^®^, version 7.08.03.110) of eligible patients based on previous agreements about the eligibility criteria, collection method of study variables, recording data, and data interpretation.

The study inclusion criteria were: (1) available xerostomia status (the subjective feeling of oral dryness, based on a question in patients’ medical history form; (2) age from 18 to 65 years; and (3) available current medication list (verified through electronic health records of the patient). The prescription list of medications was checked using the Anatomical Therapeutic Chemical (ATC) classification to avoid the underestimation of medication utilization based on self-reports by the patients. The exclusion criteria were the following: (1) Sjögren’s syndrome or other (autoimmune) salivary diseases affecting the salivary glands; (2) history of head and neck radiation as well as radioiodine treatment; and (3) prescribed cholinergic agonists.

Sample size calculation resulted in a minimum number of 233 cases as well as controls, which achieves 80% power to detect the 0.04 non-inferiority difference between the two groups at a significance level of 0.05. We extracted the maximum available data within the EIOH Axium electronic system, which exceeded the minimally sufficient sample size. Patients with recorded xerostomia status were eligible to be selected as cases. The dental charts of 649 adult patients who answered “Yes” to the question, “Do you have dry mouth?” were included for data collection. A gender- and an age-matched group of 649 adult patients who responded “No” to the same question were selected randomly and included as controls in the study. The caries status of the patients was obtained from electronic dental charts. We used the DMFT—decayed (D), missing (M), or filled (F)—tooth index to assess cumulative caries experience retrospectively. Previous studies examining xerostomia frequently used the DMFT index to assess the caries rates and reported an association with the severity of xerostomia [26,27,28]. In addition, other oral health data were collected as the total number of teeth, status of edentulism, use of dentures, smoking, and dry mouth treatment.

Anticholinergic exposure was quantified as the cumulative effect of anticholinergic drugs using the Anticholinergic Drug Scale (ADS). ADS is an expert opinion-derived risk scale based on a radioligand assay to measure in vitro anticholinergic activity of anticholinergic drugs. We calculated the ADS score of each participant through the modified ADS method [29], which consists of an updated and dose-weighted list of 536 medications; 419 medications ranked 0 with low potency, and 117 medications have a numerical ranking between 1 (potentially anticholinergic) and 3 (markedly anticholinergic).

### Statistical Analysis

Descriptive statistical analysis was completed to report means and standard deviations of age, sex, smoking, edentulism, total number of medications, total number of anticholinergic drugs, and ADS. SPSS (version 28, IBM, Chicago, IL, USA) was used to analyze the data. Frequency tables were created for all the categorical and dichotomous variables distinguishing patients with and without xerostomia status and examined using the Pearson’s Chi-squared test. Mean and standard deviation were calculated for the continuous variables. The normality of the data was analyzed using the Shapiro-Wilk test. Comparisons were made between cases and controls using the Wilcoxon Signed Rank test. The impact of DMFT, total number of medications, total number of anticholinergic medications, and ADS on xerostomia status was analyzed using binary logistic regression. We considered potential confounders and covariates and included demographic information such as age and race, medical conditions, allergies, sleep apnea, total number and type of medications, and total number of anticholinergic medications. The statistical level for significance was set at 0.05 for all the tests.

## 3. Results

Demographics, summarized in Table 1, showed that the study population broadly represented the EIOH community regarding race and ethnicity. The matching variables defined a similar proportion of sex and age. The case and control groups showed statistically different racial distributions.

The self-reported xerostomia rate in women was approximately three times higher than that in men. We evaluated the distribution of factors that are often associated with xerostomia. Smoking, diabetes, and sleep apnea showed a statistically significant higher prevalence in the xerostomia group compared to the controls. Denture wearing was associated with xerostomia status, although complete edentulism appeared with similar distribution in both study groups. The examination of caries statuses is summarized in Table 1; the mean value for all components—number of decayed, missing, and filled teeth—was significantly higher in patients who self-reported on xerostomia.

**Figure 1:**
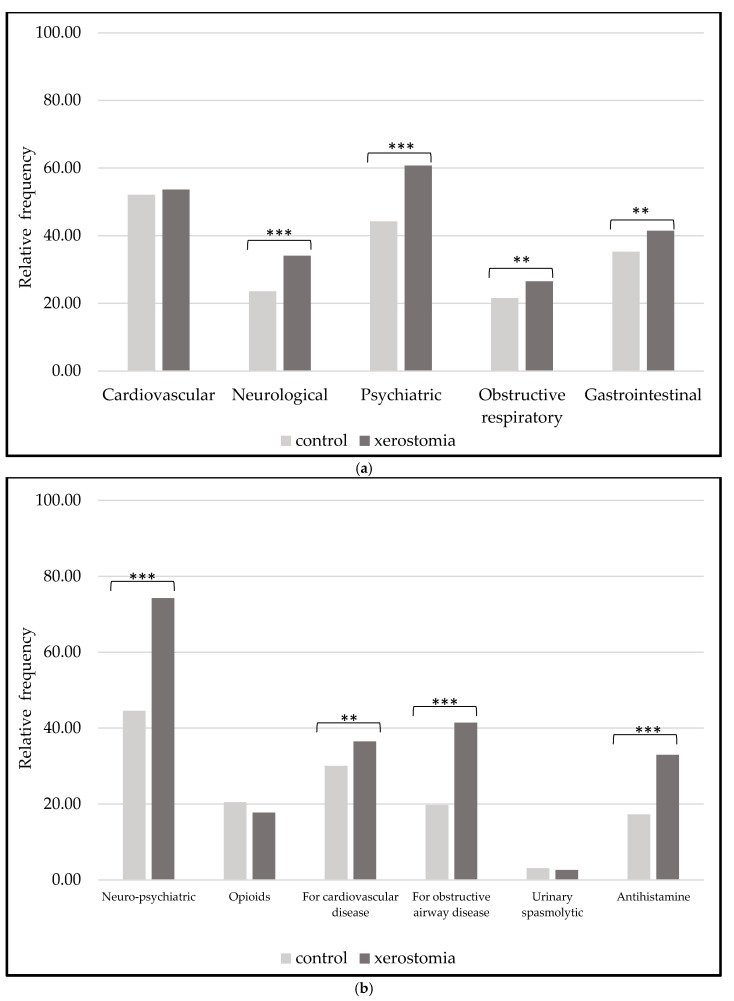
(**a**) Occurrence of medical conditions often treated with anticholinergic medications in the case and control groups. (**b**) Most frequent classes of medications with anticholinergic properties (χ^2^ test comparisons with ** *p*-value < 0.05 and *** < 0.001).

The observed frequencies of various diseases and medical conditions in which anticholinergic medications are most often prescribed are summarized in Figure 1.

Statistically significant differences in neurological, psychiatric, obstructive respiratory, and gastrointestinal diseases show higher frequencies in the xerostomia group. We noticed differences in anticholinergic medication usage (Figure 1). Patients with xerostomia (mean of the total number of anticholinergic medications 4.95 ± 3.38) were taking significantly more anticholinergic medications for neuro-psychiatric and cardiovascular diseases, as well as for obstructive airway (such as asthma or chronic obstructive pulmonary) diseases compared to the controls (mean of the total number of anticholinergic medications 2.85 ± 2.83). The utilization of antihistamines reflected a significantly higher rate among xerostomia patients. Anticholinergics in opioid and urinary spasmolytic categories were evenly distributed among the xerostomia and control groups. Comparisons of the anticholinergic burden measured in ADS resulted in patients in the xerostomia group with significantly higher anticholinergic scores. While low scores (Table 2) (ADS < 3) were more frequent within the control population (mean of 1.89 ± 2.45), high scores (ADS > 3) were calculated within 52.8% of the xerostomia group (mean of 3.26 ± 2.81).

The occurrence of polypharmacy (taking at least five prescription medications) was examined in stratified age groups in Table 3.

**Figure 2:**
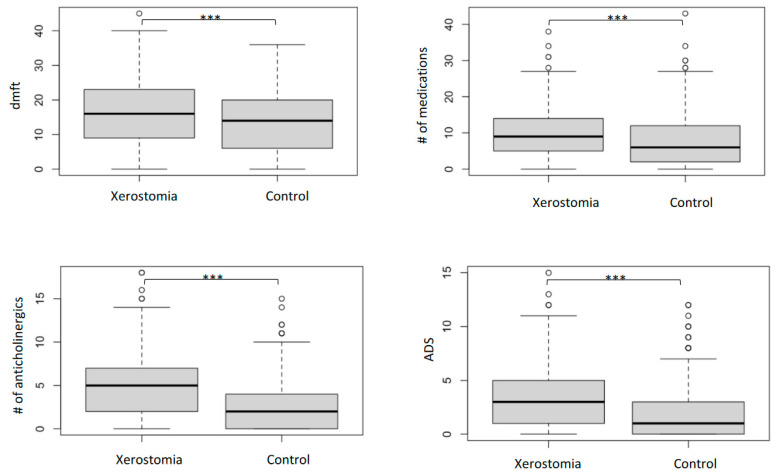
DMFT, number of medications, number (#) of anticholinergic medications, and ADS in the xerostomia and control groups. (Mann-Whitney U tests, boxplots represent the median and quartiles, *** *p*-value < 0.001).

In the youngest age group (18–35), polypharmacy was two times more frequent, and anticholinergic polypharmacy was three times more frequent in patients with xerostomia than in those without it. Similarly, we found a significantly higher proportion of polypharmacy and anticholinergic polypharmacy in the younger and middle-aged groups. A significant difference was detected in DMFT between patients with and without xerostomia (Figure 2) using a *t*-test, *t*(1202) = 4.16, *p* < 0.001. The mean DMFT for patients with and without xerostomia was 16.02 + 9.50 and 13.83 + 8.83, respectively, with a significant difference of 2.20 DMFTs (95% CI: 3.23, 1.16).

A binary logistic regression (Table 4) was performed to ascertain the effects of DMFT, the total number of medications, the total number of AC medications, and the ADS burden on xerostomia status (Y/N). The model was statistically significant, *χ*^2^(4, *n* = 1298) = 136.09, *p* < 0.001. The Hosmer-Lemeshow test showed that the model fitted the data well, *p* = 0.338. DMFT, the total number of medications, and the total number of AC medications are significant predictors. Accordingly, adding each AC anticholinergic medication increases the odds of having xerostomia by 50% (OR = 1.50), and each DMFT increases the odds of having xerostomia by 2% (OR = 1.02).

## 4. Discussion

Our case–control clinical study with a sample of 649 patients with xerostomia and 649 age- and gender-matched controls examined the relationship between anticholinergic medication-induced xerostomia and caries status among adults between 18 and 65 years of age. Our findings indicated significant differences in anticholinergic medication burden between the case group of adults with xerostomia and the control group. Polypharmacy and anticholinergic polypharmacy were more frequently observed in patients with xerostomia. Individuals with xerostomia had higher caries rates and a higher level of anticholinergic exposure from medications compared to their age and gender-matched controls without xerostomia. Logistic regression analysis confirmed that DMFT, the total number of AC medications, and the ADS burden significantly affected the xerostomia status. Comparisons between case and control groups revealed statistical differences in risk factors, such as smoking, diabetes, and sleep apnea, as well as in the utilization of anticholinergic medications. We found that increased caries prevalence and a higher ADS score are associated with increased xerostomia risk.

Our study reveals a statistically different caries status among younger or middle-aged individuals with and without xerostomia related to medication number and anticholinergic burden. In a previous pilot study investigating middle-aged xerostomia patients, we reported increased caries prevalence, reflecting the estimated level of anticholinergic medication exposure. Anticholinergic medications can severely compromise salivary secretion and reduce saliva’s protective actions. Clinical studies have shown that a reduced saliva flow rate increases caries experience [6]. Saliva contains considerable amounts of calcium and phosphate, which decrease hydroxyapatite’s solubility, the tooth substance’s primary component. The salivary bicarbonate increases the pH and buffer capacity of the saliva. It, therefore, plays a role in inhibiting tooth demineralization caused either by bacterial acids or free acids in drinks and foods by decreasing the solubility of hydroxyapatite [30]. Carious lesions in xerostomia occur with a rapid onset and progression, and they are frequently seen in the cervical areas of teeth. Denture wearers were more commonly identified in the xerostomia group, which was not yet reported in the literature.

Anticholinergics are the largest group of xerostomia-inducing medications, interfering with the parasympathetic signaling responsible for saliva secretion [13,31]). Over 600 medications have the potency to inhibit the secretion of the salivary glands, which are the most sensitive target organs of anticholinergics. The growing tendencies in medication usage show that polypharmacy increased by 70% [14], and anticholinergic exposure from medications has grown significantly [32]. Anticholinergic medications are common among older adults, and the prevalence of xerostomia was statistically significantly higher in patients using anticholinergic medications [8]. Moreover, a meta-analysis of 26 studies [33] concluded that the risk for xerostomia increases upon combining those medications.

Polypharmacy, expressed in total or anticholinergic medications, was two or three times higher, respectively, in the xerostomia cohorts of young ages. Our data are confirmed by an earlier investigation [34], which reported a significant association between increasing xerostomia risk and the number of medications used. The total number of medications in our xerostomia group was comparable to a recent publication of patients over 65 [35], which could be explained by the compromised general health status of the patients with medication-induced xerostomia in our study. We collected data from our safety-net providers committed to community service and equitable health care; therefore, a significant percentage of our patient population is medically complex and underserved. A recent study [36], mainly including men of 20–99 years with xerostomia diagnosis, also revealed a correspondingly high polypharmacy rate (75%) when compared to our study (80%). Additionally, the authors reported a strikingly similar average number of medications as we found in our xerostomia group.

We measured ADS to explore the anticholinergic burden, but comparable available data about the anticholinergic exposure of the younger population are minimal. Less than half of the control patients scored ADS = 0 compared to 20% of the xerostomia cases. The majority of patients in both groups had a countable score of ADS. High scores above three pose a severe anticholinergic burden [37], and the risk for decreased saliva secretion was reported as twice as prevalent among patients with xerostomia. The average ADS in our case and control groups exceeded the published scores. A survey by Tiisanoja et al. [22] of middle-aged individuals showed a 14% xerostomia prevalence rate, and only 8% had ADS = 1. Kersten et al. [38] estimated an ADS > 6 in 13% of older adults, a prevalence that falls between our case and cohort groups.

The most commonly dispensed anticholinergic medications, reflecting the most frequent underlying medical conditions, were antidepressants, antipsychotics, opioids, antihistamines, and medications used for cardiovascular and obstructive airway diseases in the xerostomia as well as in the control group. The magnitude and order of the utilization of the most consumed drug classes were consistent with the study among more than 30,000 middle-aged participants by Ziad et al. [39]. Our findings are also supported by the most recent available data in xerostomia patients, published by Fortuna et al. [40] described that taking more than one anticholinergic medication results in a more significant negative impact on the saliva flow. The “Xeromed” analysis was based on a cross-sectional, multicenter study of 1144 patients. Although the mean age in our study was ten years younger, the gender ratio reflected similarities in favor of women. Additionally, the most frequently used anticholinergics were ranked in a comparable order, including opioids, antidepressants, benzodiazepines, respiratory agents, and antihypertensives. Younger adult patients in our study were most frequently taking neuropsychiatric medications, similar to a previous observation [41] that medication consumption showed the most significant increase among adolescents in the class for the central nervous system.

Besides the xerogenic effect of medications, other systemic and risk factors play a role in the development of xerostomia. Smokers are likely to have lower saliva flow [42], and our analysis confirmed that the smoking rate was indeed higher among individuals with xerostomia. Comorbidities with potential salivary effects, such as diabetes and sleep apnea, followed the same tendency. It should be added that stress is thought to be a possible cause of xerostomia in younger adults [43]. Other mechanisms underlying xerostomia cases may include sympathomimetic effects, topical effects of inhaled medications, dehydration, vasoconstriction in salivary glands, alterations in electrolyte and fluid balance, and changes in saliva composition.

Limitations owing to the retrospective nature of this study did not allow further investigation into other caries risk factors. Dental caries is a multifactorial disease heavily influenced by oral health profiles, behavioral changes, mental or physical disabilities, diet, and oral microbiota. Information was unavailable about caries disease risk factors and data on confounding variables such as periodontal health, gingival or plaque index, ability to brush teeth, and daily home-based dental care. Temporal aggregation of carious processes and tooth loss for various reasons over the time elapsed prior to the recording of DMFT should be considered. Xerostomia diagnosis was established on single-term self-reports, in contrast to the objective measure of oral dryness using the saliva flow rates. The community cohort determined the characteristics of the study population at our dental clinics. Gender representation in our study groups reflected the commonly observed and published gender difference in xerostomia as the cohorts were retrospectively established. We estimated the anticholinergic burden using the ADS scale (validated previously in the oral health context), although the gold standard assessment would require a serum level measurement. Therefore, a prospective study is warranted to confirm this study’s findings and establish the predictive risk for medication-induced xerostomia.

The present study established associations between xerostomia and anticholinergic burden, and anticholinergic polypharmacy, as well as caries experience. Based on 1298 patients, our data demonstrate the importance of assessing the clinical signs of xerostomia and reviewing medication history when treating dental patients, even if they belong to non-geriatric age groups. In the context of preventing xerostomia-related caries and oral health damages, the study brought into focus the evaluation of the anticholinergic burden. Although regular monitoring of saliva secretion is seldom completed during appointments, personalized screening based on medication history and evaluating the anticholinergic burden should be integrated into the dental treatment planning for the non-geriatric population.

## Figures and Tables

**Table 1:** Demographics and descriptive analysis of the dental status and caries risk factors of *n* = 1298 adults under age 65 (Chi-squared, Wilcoxon Signed Rank tests).

	Xerostomia (*n* = 649)	Control (*n* = 649)	
Age (years)			
Mean	47.53	47.32	
SD	11.78	12.08	
Gender (%)			
Female	72.63	72.26	
Male	27.37	27.74	
Race (%)			*p* < 0.001
White	60.40	43.91	
Black	17.10	24.65	
NA	22.50	31.44	
Smoking (%)	45.92	33.89	*p* < 0.001
Diabetes (%)	22.68	16.48	*p* < 0.05
Sleep apnea (%)	10.79	4.47	*p* < 0.001
Wearing dentures (%)	55.47	16.79	*p* < 0.001
Complete edentulous (%)	10.48	8.01	
D; number of decayed teeth			
Mean	1.86	1.5	*p* < 0.001
SD	3.35	2.76	
M; number of missing teeth			
Mean	7.65	6.16	*p* < 0.001
SD	8.89	7.50	
F; number of filled teeth			
Mean	6.62	6.32	*p* < 0.001
SD	5.45	5.47	

**Table 2:** Prevalence of low, medium, and high anticholinergic drug scores (ADS).

		Xerostomia (*n* = 649)		Control (*n* = 649)	
ADS		Number of patients	Cumulative frequency	Number of patients	Cumulative frequency
ADS < 3	low	287	45.6	421	70.2
3 ≤ ADS ≤6	medium	215	79.7	121	90.4
ADS > 6	high	128	100	58	100

**Table 3:** Proportions of polypharmacy and anticholinergic polypharmacy. Comparison of observed frequencies in each age category was completed using the Chi-squared test (*p*-value).

Age Group	18–35 Years		36–50 Years		51–65 Years	
	Control (*n* = 121)	Xerostomia (*n* = 122)	Control (*n* = 192)	Xerostomia (*n* = 203)	Control (*n* = 337)	Xerostomia (*n* = 324)
Polypharmacy	44	81	117	170	216	280
	*p* = 0.007		*p* = 0.042		*p* = 0.015	
Anticholinergic polypharmacy	13	44	47	118	98	185
	*p* < 0.001		*p* < 0.001		*p* < 0.001	

**Table 4:** Results of binary logistic regression.

Term	Coefficient	SE Coef	95% CI	Z-Value	*p*-Value	VIF
Constant	−0.842	0.140	(−1.116, −0.569)	−6.03	0.000	
Total number of medications	−0.112	0.0186	(−0.148, −0.075)	−5.99	0.000	3.45
Number of anticholinergics	0.389	0.046	(0.299, 0.480)	8.48	0.000	4.47
DMFT (D + M + F)	0.0188	0.007	(0.005, 0.033)	2.67	0.008	1.03
ADS	0.0272	0.037	(−0.045, 0.099)	0.74	0.461	2.14
			**Odds Ratio**		**95% CI**	
Total number of medications			0.894		(0.862, 0.928)	
Number of anticholinergics			1.477		(1.349, 1.616)	
DMFT (D + M + F)			1.019		(1.005, 1.033)	
ADS			1.028		(0.956, 1.105)	
				**Wald Test**		
**Source**			**DF**	**Chi-Squared**		***p*-Value**
Regression			4	136.09		0.000
Total number of medications			1	35.89		0.000
Number of anticholinergics			1	71.85		0.000
DMFT (D + M + F)			1	7.12		0.008
ADS			1	0.54		0.461

Abbreviations: SE Coef, standard error of the coefficient; CI, confidence interval; VIF, variance inflation factor, DMFT, decayed, missed, filled tooth; ADS, anticholinergic drug scale; DF, degrees of freedom.

## Data Availability

Data available on request due to restrictions of Health Insurance Portability and Accountability Act (HIPAA). The data presented in this study are available on request from the corresponding author.

## References

[1] Ohara Y., Hirano H., Yoshida H., Obuchi S., Ihara K., Fujiwara Y., Mataki S. (2013). Prevalence and factors associated with xerostomia and hyposalivation among community-dwelling older people in Japan. Gerodontology.

[2] Villa A., Nordio F., Gohel A. (2016). A risk prediction model for xerostomia: A retrospective cohort study. Gerodontology.

[3] Friedlander A.H., Norman D.C. (2002). Late-life depression: Psychopathology, medical interventions, and dental implications. Oral Surg. Oral Med. Oral Pathol. Oral Radiol. Endod..

[4] Agostini B.A., Cericato G.O., da Silveira E.R., Nascimento G.G., dos Santos Costa F., Thomson W.M., Demarco F.F. (2018). How Common is Dry Mouth? Systematic Review and Meta-Regression Analysis of Prevalence Estimates. Braz. Dent. J..

[5] Da Silva L., Kupek E., Peres K.G. (2017). General health influences episodes of xerostomia: A prospective population-based study. Community Dent. Oral Epidemiol.

[6] Bardow A., Nyvad B., Nauntofte B. (2001). Relationships between medication intake, complaints of dry mouth, salivary flow rate and composition, and the rate of tooth demineralization in situ. Arch. Oral Biol..

[7] Hu K.-F., Chou Y.-H., Wen Y.-H., Hsieh K.-P., Tsai J.-H., Yang P., Yang Y.-H., Lin C.-H.R. (2016). Antipsychotic medications and dental caries in newly diagnosed schizophrenia: A nationwide cohort study. Psychiatry Res..

[8] Thomson W.M., Lawrence H.P., Broadbent J.M., Poulton R. (2006). The impact of xerostomia on oral-health-related quality of life among younger adults. Health Qual. Life Outcomes.

[9] Aliko A., Wolff A., Dawes C., Aframian D., Proctor G., Ekström J., Narayana N., Villa A., Sia Y.W., Joshi R.K. (2015). World Workshop on Oral Medicine VI: Clinical implications of medication-induced salivary gland dysfunction. Oral Surg. Oral Med. Oral Pathol. Oral Radiol..

[10] Jager D.H.J., Bots C.P., Forouzanfar T., Brand H.S. (2018). Clinical oral dryness score: Evaluation of a new screening method for oral dryness. Odontology.

[11] Wolff A., Zuk-Paz L., Kaplan I. (2008). Major salivary gland output differs between users and non-users of specific medication categories. Gerodontology.

[12] Mathews S.A., Kurien B.T., Scofield R.H. (2008). Oral manifestations of sjögren’s syndrome. J. Dent. Res..

[13] Arany S., Kopycka-Kedzierawski D.T., Caprio T.V., Watson G.E. (2021). Anticholinergic medication: Related dry mouth and effects on the salivary glands. Oral Surg. Oral Med. Oral Pathol. Oral Radiol..

[14] Kantor E.D., Rehm C.D., Du M., White E., Giovannucci E.L. (2016). Trends in Dietary Supplement Use among US Adults From 1999–2012. JAMA.

[15] Thomson W.M. (2015). Dry mouth and older people. Aust. Dent. J..

[16] Halli-Tierney A.D., Scarbrough C., Carroll D. (2019). Polypharmacy: Evaluating Risks and Deprescribing. Am. Fam. Physician.

[17] Menditto E., Gimeno-Miguel A., Juste A.M., Poblador-Plou B., Aza-Pascual-Salcedo M., Orlando V., Rubio F.G., Torres A.P. (2019). Patterns of multimorbidity and polypharmacy in young and adult population: Systematic associations among chronic diseases and drugs using factor analysis. PLoS ONE.

[18] Ferrari A., Baraldi C., Licata M., Rustichelli C. (2018). Polypharmacy Among Headache Patients: A Cross-Sectional Study. CNS Drugs.

[19] Field E., Fear S., Higham S., Ireland R., Rostron J., Willetts R.M., Longman L. (2001). Age and medication are significant risk factors for xerostomia in an English population, attending general dental practice. Gerodontology.

[20] Tollefson G.D., Montague-Clouse J., Lancaster S.P. (1991). The relationship of serum anticholinergic activity to mental status performance in an elderly nursing home population. J. Neuropsychiatry.

[21] Tune L.E., Damlouji N.F., Holland A., Gardner T.J., Folstein M.F., Coyle J.T. (1981). Association of postoperative delirium with raised serum levels of anticholinergic drugs. Lancet.

[22] Tiisanoja A., Syrjälä A.-M., Kullaa A., Ylöstalo P. (2020). Anticholinergic Burden and Dry Mouth in Middle-Aged People. JDR Clin. Transl. Res..

[23] Carnahan R.M., Lund B.C., Perry P.J., Pollock B.G., Culp K.R. (2006). The Anticholinergic Drug Scale as a Measure of Drug-Related Anticholinergic Burden: Associations with Serum Anticholinergic Activity. J. Clin. Pharmacol..

[24] Kersten H., Wyller T.B., Molden E. (2014). Association between inherited cyp2d6/2c19 phenotypes and anticholinergic measures in elderly patients using anticholinergic drugs. Ther. Drug Monit..

[25] Tiisanoja A., Syrjala A.M., Anttonen V., Ylostalo P. (2021). Anticholinergic burden, oral hygiene practices, and oral hygiene status-cross-sectional findings from the northern finland birth cohort 1966. Clin. Oral. Investig..

[26] Chapuis J., Siu-Paredes F., Pavageau C., Amador G., Rude N., Denis F. (2020). Anticholinergic drugs and oral health-related quality of life in patients with schizophrenia: A pilot study. Transl. Neurosci..

[27] Janssens B., Vanobbergen J., Petrovic M., Jacquet W., Schols J.M., De Visschere L. (2018). The impact of a preventive and curative oral healthcare program on the prevalence and incidence of oral health problems in nursing home residents. PLoS ONE.

[28] Lexomboon D., Tan E.C., Höijer J., Garcia-Ptacek S., Eriksdotter M., Religa D., Fastbom J., Johnell K., Sandborgh-Englund G. (2018). The Effect of Xerostomic Medication on Oral Health in Persons with Dementia. J. Am. Med. Dir. Assoc..

[29] Durán C.E., Azermai M., Stichele R.H.V. (2013). Systematic review of anticholinergic risk scales in older adults. Eur. J. Clin. Pharmacol..

[30] Bardow A., Moe D., Nyvad B., Nauntofte B. (1999). The buffer capacity and buffer systems of human whole saliva measured without loss of CO_2_. Arch. Oral Biol..

[31] Scully C. (2003). Drug effects on salivary glands: Dry mouth. Oral. Dis..

[32] Kachru N., Carnahan R.M., Johnson M.L., Aparasu R.R. (2015). Potentially Inappropriate Anticholinergic Medication Use in Community-Dwelling Older Adults: A National Cross-Sectional Study. Drugs Aging.

[33] Tan E.C.K., Lexomboon D., Englund G.S., Haasum Y., Johnell K. (2018). Medications That Cause Dry Mouth as an Adverse Effect in Older People: A Systematic Review and Metaanalysis. J. Am. Geriatr. Soc..

[34] Nederfors T., Isaksson R., Mornstad H., Dahlof C. (1997). Prevalence of perceived symptoms of dry mouth in an adult Swedish population--relation to age, sex and pharmacotherapy. Community Dent. Oral Epidemiol..

[35] Minagi H.O., Yamanaka Y., Nohara K., Ikai K., Sakai T. (2021). Analysis of medication-induced xerostomia in elderly Japanese patients. Clin. Oral Investig..

[36] Marcott S., Dewan K., Kwan M., Baik F., Lee Y.-J., Sirjani D. (2020). Where Dysphagia Begins: Polypharmacy and Xerostomia. Fed. Pract. Health Care Prof. VA DoD PHS.

[37] Tiisanoja A., Syrjälä A.-M., Komulainen K., Lampela P., Hartikainen S., Taipale H., Knuuttila M., Ylöstalo P. (2018). Anticholinergic burden and dry mouth among Finnish, community-dwelling older adults. Gerodontology.

[38] Kersten H., Molden E., Willumsen T., Engedal K., Wyller T.B. (2013). Higher anticholinergic drug scale (ADS) scores are associated with peripheral but not cognitive markers of cholinergic blockade. Cross sectional data from 21 Norwegian nursing homes. Br. J. Clin. Pharmacol..

[39] Ziad A., Olekhnovitch R., Ruiz F., Berr C., Bégaud B., Goldberg M., Zins M., Mura T. (2018). Anticholinergic drug use and cognitive performances in middle age: Findings from the constances cohort. J. Neurol. Neurosurg. Psychiatry.

[40] Fortuna G., Whitmire S., Sullivan K., Alajbeg I., Andabak-Rogulj A., Pedersen A.M.L., Vissink A., di Fede O., Aria M., Jager D.J. (2022). Impact of medications on salivary flow rate in patients with xerostomia: A retrospective study by the Xeromeds Consortium. Clin. Oral Investig..

[41] Lagerberg T., Molero Y., D’Onofrio B.M., de la Cruz L.F., Lichtenstein P., Mataix-Cols D., Rück C., Hellner C., Chang Z. (2019). Antidepressant prescription patterns and CNS polypharmacy with antidepressants among children, adolescents, and young adults: A population-based study in Sweden. Eur. Child Adolesc. Psychiatry.

[42] Rad M., Kakoie S., Brojeni F.N., Pourdamghan N. (2010). Effect of Long-term Smoking on Whole-mouth Salivary Flow Rate and Oral Health. J. Dent. Res. Dent. Clin. Dent. Prospect..

[43] Setia S., Pannu P., Gambhir R., Galhotra V., Ahluwalia P., Sofat A. (2014). Correlation of oral hygiene practices, smoking and oral health conditions with self perceived halitosis amongst undergraduate dental students. J. Nat. Sci. Biol. Med..

